# A Serious Game Designed to Promote Safe Behaviors Among Health Care Workers During the COVID-19 Pandemic: Development of “Escape COVID-19”

**DOI:** 10.2196/24986

**Published:** 2020-12-03

**Authors:** Mélanie Suppan, Gaud Catho, Tomás Robalo Nunes, Valérie Sauvan, Monique Perez, Christophe Graf, Didier Pittet, Stephan Harbarth, Mohamed Abbas, Laurent Suppan

**Affiliations:** 1 Division of Anesthesiology Department of Anesthesiology, Clinical Pharmacology, Intensive Care and Emergency Medicine University of Geneva Hospitals and Faculty of Medicine Geneva Switzerland; 2 Infection Control Program WHO Collaborating Centre on Patient Safety University of Geneva Hospitals and Faculty of Medicine Geneva Switzerland; 3 Infectious Diseases Department Hospital Garcia de Orta EPE Almada Portugal; 4 Department of Rehabilitation and Geriatrics Geneva University Hospital Geneva Switzerland; 5 Division of Emergency Medicine Department of Anesthesiology, Clinical Pharmacology, Intensive Care and Emergency Medicine University of Geneva Hospitals and Faculty of Medicine Geneva Switzerland

**Keywords:** COVID-19, transmission, serious games, infection prevention, health care workers, SARS-COV-2, infectious disease, safety, behavior, hospital

## Abstract

**Background:**

As many countries fear and even experience the emergence of a second wave of COVID-19, reminding health care workers (HCWs) and other hospital employees of the critical role they play in preventing SARS-CoV-2 transmission is more important than ever. Building and strengthening the intrinsic motivation of HCWs to apply infection prevention and control (IPC) guidelines to avoid contaminating their colleagues, patients, friends, and relatives is a goal that must be energetically pursued. A high rate of nosocomial infections during the first COVID-19 wave was detected by IPC specialists and further cemented their belief in the need for an engaging intervention that could improve compliance with COVID-19 safe behaviors.

**Objective:**

Our aim was to develop a serious game that would promote IPC practices with a specific focus on COVID-19 among HCWs and other hospital employees.

**Methods:**

The first 3 stages of the SERES framework were used to develop this serious game. A brainswarming session between developers and IPC specialists was used to identify the target audience and acquisition objectives. Nicholson’s RECIPE mnemonic (reflection, engagement, choice, information, play, exposition) for meaningful gamification was used to guide the general design. A common and simple terminology was used to suit the broad target audience. The game was tested on various platforms (smartphones, tablets, laptops, desktop computers) by different users during each development loop and before its final release.

**Results:**

The game was designed to target all hospital staff who could be in direct contact with patients within the Geneva University Hospitals. In total, 10 acquisition objectives were defined by IPC specialists and implemented into the game according to the principles of meaningful gamification. A simple storyboard was first created using Microsoft PowerPoint and was progressively refined through multiple iteration loops. Articulate Storyline was then used to create two successive versions of the actual game. In the final version, a unique graphic atmosphere was created with help from a professional graphic designer. Feedback mechanisms were used extensively throughout the game to strengthen key IPC messages.

**Conclusions:**

The SERES framework was successfully used to create “Escape COVID-19,” a serious game designed to promote safe IPC practices among HCWs and other hospital employees during the COVID-19 pandemic. This game can be obtained free of charge for research and educational purposes. A SCORM (shareable content object reference model) package is available to facilitate results and completion tracking on most current learning management systems.

## Introduction

### Background and Importance

As the COVID-19 death toll continues to rise [1] and many countries fear or are experiencing the emergence of a second wave [2-4], avoiding disease transmission is becoming increasingly important. Health care workers (HCWs) and other hospital employees, including medical, nursing, and administrative and support staff, are at high risk of contamination [5-7]. The actual rate of COVID-19 infection among HCWs is unknown and may vary significantly from one country to another. Recent studies have shown that as many as 20% of asymptomatic HCWs had serological evidence of having been exposed to SARS-CoV-2, the virus responsible for COVID-19 [8]. Inadequate application of infection prevention and control (IPC) procedures by HCWs and other hospital employees can lead to the contamination of their colleagues, patients, and even visitors [9]. Improper handling of personal protective equipment (PPE) can also lead to self-contamination [10,11].

As the pandemic draws out, lassitude and conspiracy theories can lead to a slackening of safety measures [12,13], including among HCWs. Moreover, some HCWs seem to lack critical knowledge regarding the modes of transmission of SARS-CoV-2 [14]. In addition, the frequent and sometimes conflicting updates pertaining to COVID-19 guidelines have led many HCWs to question their applicability and protective value [15]. This can lead to a lack of commitment, one of the barriers that negatively impacts the implementation of measures necessary to prevent SARS-CoV-2 transmission [16]. Regular channels used to spread IPC messages might therefore fail in the current context.

Nevertheless, building and strengthening the intrinsic motivation of HCWs to rigorously apply IPC guidelines and to do their very best to avoid contaminating their patients, colleagues, friends, and relatives is a goal that must be energetically pursued [17]. Apart from the significant morbidity and mortality associated with COVID-19 infection [18], the economic burden is also worth considering. Indeed, the median costs incurred for a single case exceed $3000 according to a recent evaluation [19].

The high rate of nosocomial infections detected during the first COVID-19 wave and the need to train a large number of people while respecting the need for physical distance require the creation of original and inspirational material to promote IPC guidelines. Serious games possess motivational properties that can enhance learner engagement and satisfaction while still delivering important messages [20].

### Objective

Our aim was to develop a serious game to promote the dissemination of COVID-19 IPC practices among HCWs and other hospital employees. By raising awareness about situations that could potentially lead to COVID-19 contamination in this key population, such a game could help decrease SARS-CoV-2 transmission between HCWs and from HCWs to patients.

## Methods

### General Design

The first 3 stages of the SERES framework were used to develop this serious game [21]. This framework has already been used successfully for the development of serious games [22] and gamified e-learning (electronic learning) modules [23]. Briefly, this framework was created to help developers design theory-driven, evidence-based serious games for health. The first stage, scientific foundations, is designed to ensure that the game is both evidence-based and theoretically driven. The target audience is identified at this stage. The second stage, design foundations, focuses on the translation of these foundations into design elements. Game development takes place in the third stage of this framework.

### Scientific Foundations

#### Target Audience

The target audience was identified through a brainswarming session [24] between the lead developer (MS) and IPC specialists (GC, TRN, VS, MP, SH, and MA). The game was developed at the request of these specialists, who were therefore included in the development process from the start.

#### Acquisition Objectives

Field observations by specialists from the Geneva University Hospitals infection control program were used to identify the acquisition objectives. We decided to use the term “acquisition objectives” rather than “learning objectives” as the main purpose of this serious game was to promote and stimulate desirable behaviors in HCWs rather than to teach them new procedures.

#### Theoretical Basis

The theoretical bases relevant to the creation of gamified content in the context of the COVID-19 pandemic were already identified in the course of a previously published project [23]. Therefore, we decided to use these bases and to reassess the relevant references. Briefly, we had searched the medical literature via PubMed (using Medical Subject Headings [MeSH] and Boolean operators) and retrieved articles based on their titles and abstracts. References from the most relevant articles were also searched manually to identify sources that could have been missed. A total of 12 articles was included through this procedure. Further details are available in our previous publication [23].

We also performed a search for articles reporting information related to SARS-CoV-2 transmission. The MeSH keywords “covid-19,” “sars-cov-2,” and “transmission” were used in combination with the Boolean operators “AND” and “OR” to retrieve potentially relevant references from MEDLINE using the PubMed engine. To avoid inducing doubts or even mistrust in HCWs [25-27], the results retrieved through this search were anaylzed to determine whether they were in line with our institutional [28] and federal [29] guidelines. Whenever conflicting information was identified, a consensus was reached with the IPC specialists to determine and use the most accurate and least confusing messages.

#### Content Validation

The scientific grounds on which this serious game relies were decided during brainswarming sessions between the developers and IPC specialists from the Geneva University Hospitals. Each iteration of the serious game was reviewed by at least 2 such specialists to ensure that the intended message was correctly and adequately conveyed by the serious game.

### Design Foundations

#### General Design and Meaningful Gamification

The scientific foundations reviewed or established during the previous stage were used to create the general design of the serious game. We used Nicholson’s RECIPE mnemonic (reflection, engagement, choice, information, play, and exposition) for meaningful gamification to guide the development of the game mechanics [30].

Arnab et al’s [31] model of transforming learning mechanics into game mechanics was adapted to suit the development of this serious game. The original model states that learning mechanics should be decided according to learning objectives and that these learning mechanics should then be translated into game mechanics. As we had decided to use the term “acquisition objectives” rather than “learning objectives,” we adapted Arnab et al’s [31] model to develop a very similar “acquisition mechanics – game mechanics” model.

#### Design Requirements

Design decisions were tailored to the target population and the COVID-19 context. Ease of access to the serious game was considered to be of paramount importance, as was the ability to rapidly capture the player’s attention and interest. True to the SERES framework, we decided to use an iterative approach to build the game. We therefore sought regular feedback from the IPC specialists as well as from potential end users and implemented the required adaptations accordingly.

#### Game Development

All the data gathered through the scientific foundation and design foundation stages were used to choose a development platform and to proceed with the development of the serious game. Acquisition objectives were reassessed, and theoretical bases were used to decide upon acquisition mechanics and game mechanics.

#### Tool Evaluation

In line with the theoretical bases, the game was tested on various platforms (smartphones, tablets, laptops, desktop computers) by different users at the end of each development loop and before its final release.

## Results

### Scientific Foundations

#### Target Audience

Early on in the development process, developers and IPC specialists agreed that the target audience should be composed of all the professionals who could be in direct contact with patients within the Geneva University Hospitals. The game was therefore designed to target many different kinds of health care professionals including physicians, nurses, physiotherapists, assistants, technicians, and therapists but also non–health care professionals. Indeed, some of these employees have close and regular contact with patients (stretcher-bearers, housekeepers, etc) and might therefore be exposed to patients or play a role in SARS-CoV-2 transmission.

#### Theoretical Basis

**Figure 1:**
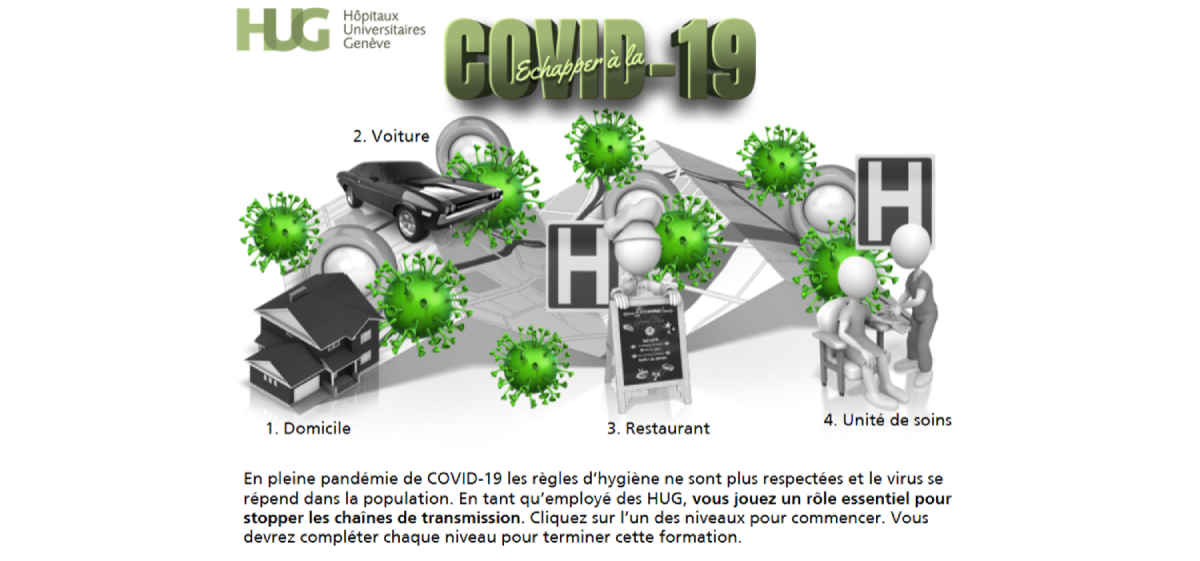
First version of the welcome screen.

**Figure 2:**
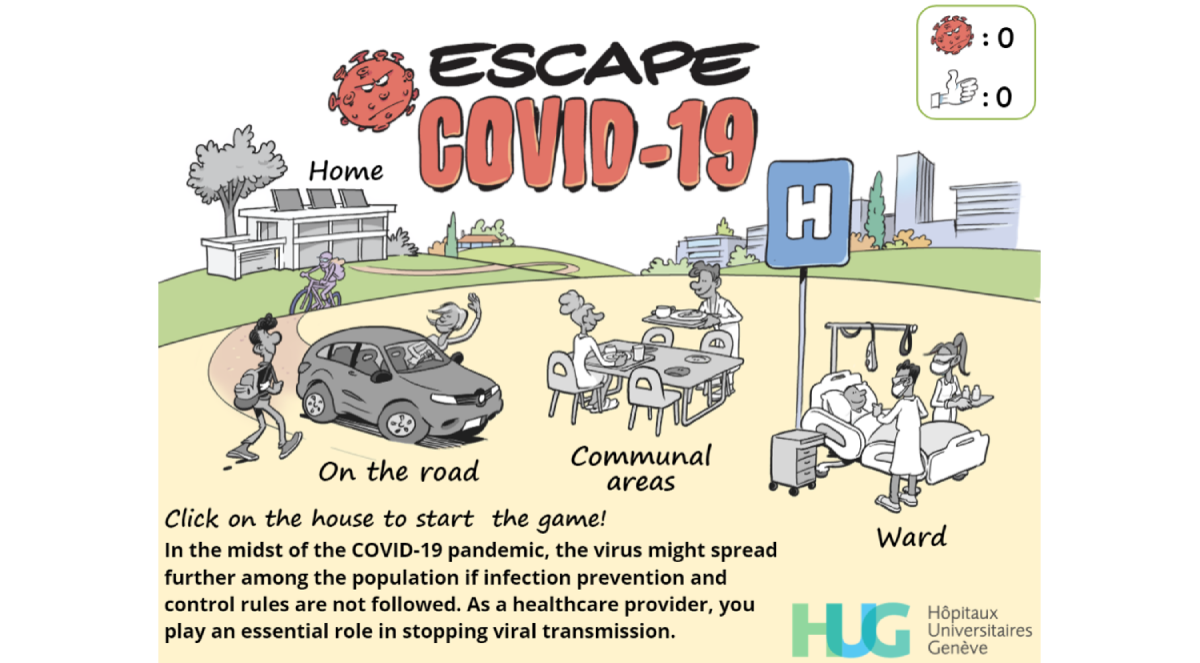
Final version of the welcome screen.

**Figure 3:**
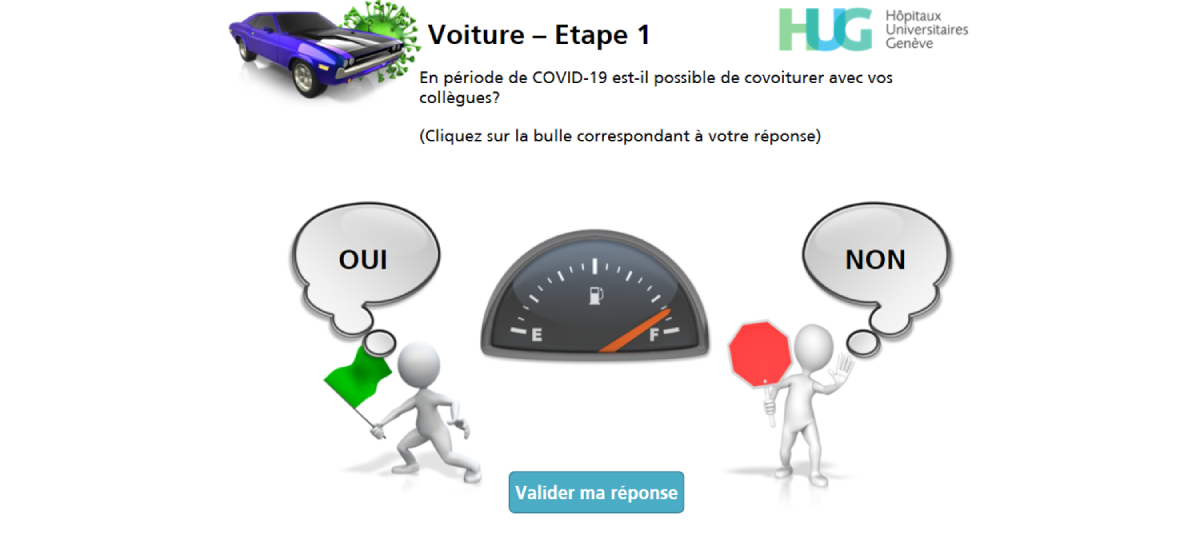
First version of a simple-choice interaction.

**Figure 4:**
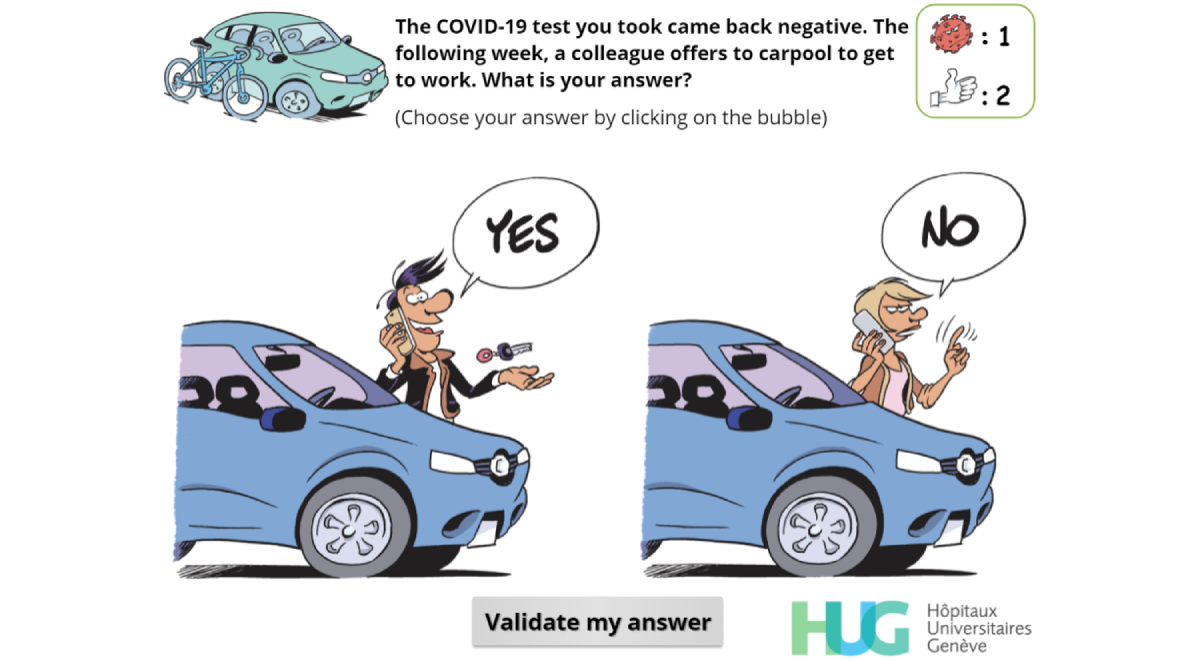
Final version of a simple-choice interaction.

**Figure 5:**
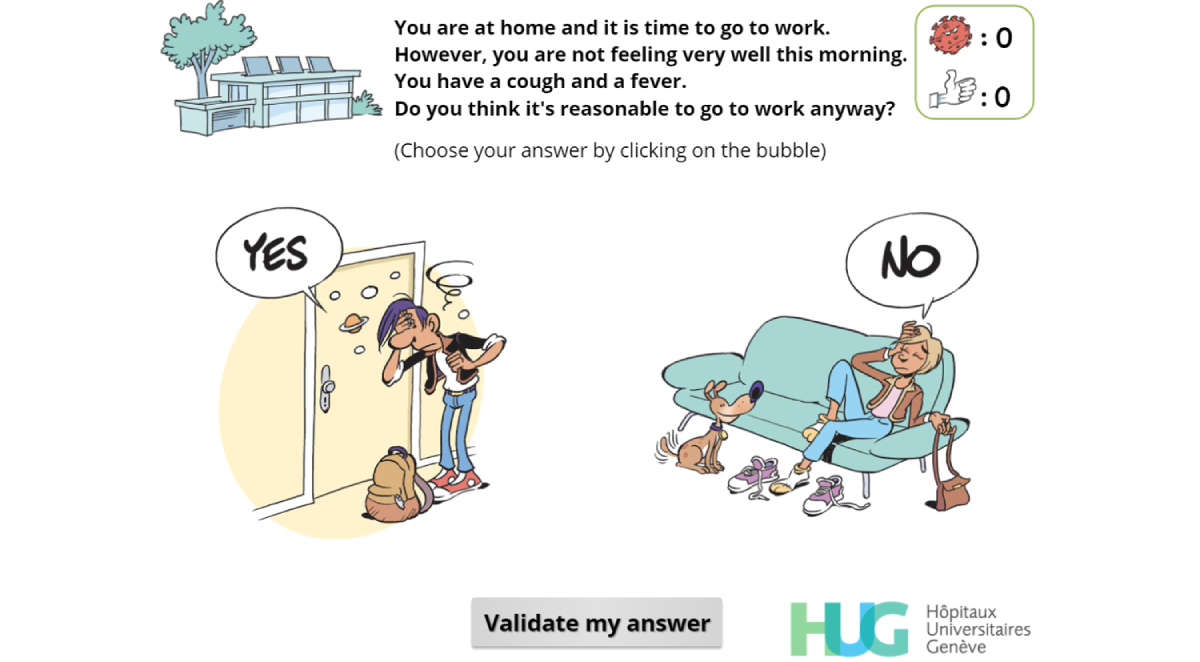
Simple-choice interaction.

**Figure 6:**
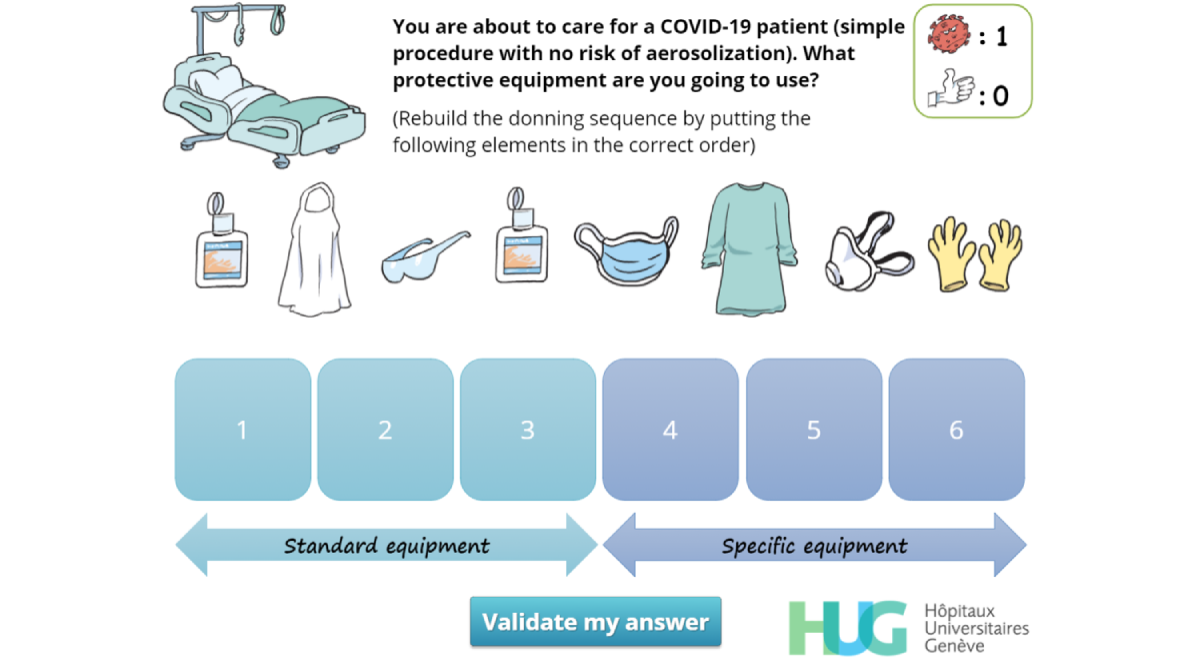
Rebuilding the donning sequence. The player must drag and drop the elements of personal protective equipment in the right order.

**Figure 7:**
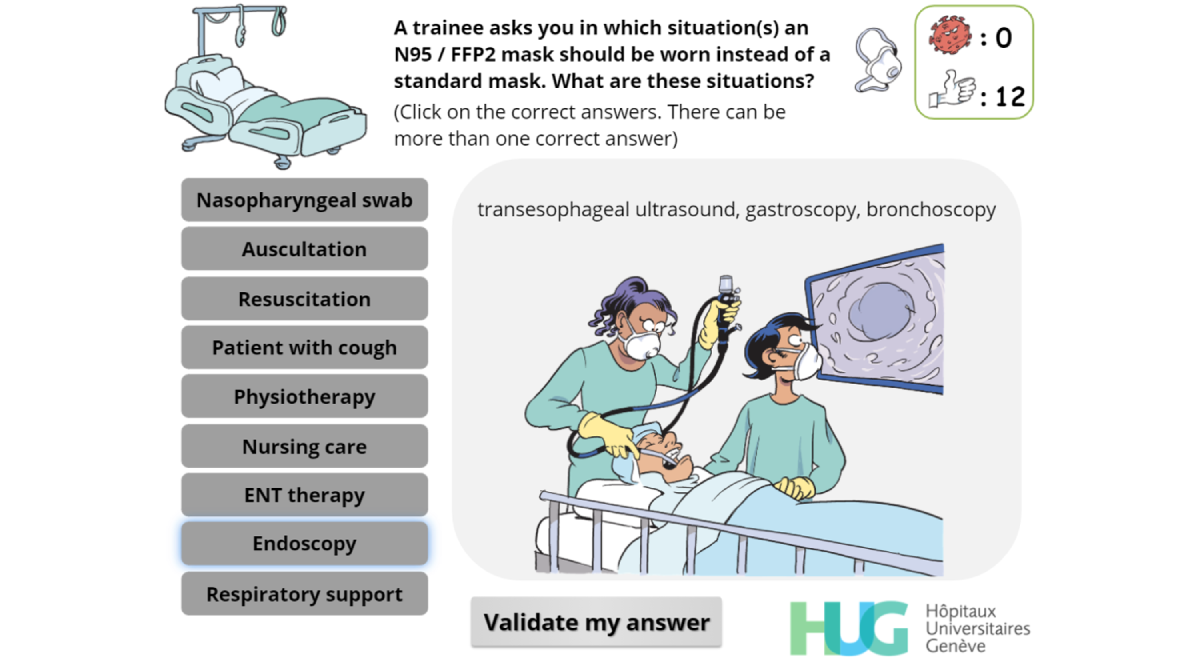
Multiple-choice interaction. The player must choose in which situations N95 masks should be worn. Illustrations change as the cursor hovers over the buttons on the left.

We used previously identified resources to establish the theoretical background [20,31-37]. These resources were extracted from Verschueren et al [22], which described the development of a serious game designed to reduce perioperative anxiety in children, and from our recent study describing the development of a gamified e-learning module that was used to teach the adequate use of PPE to prehospital personnel in the context of the COVID-19 pandemic [23]. Moreover, we sought to include all 6 elements of Nicholson’s meaningful gamification [30]. These elements are listed below and follow Nicholson’s RECIPE mnemonic:

- The “reflection” element seeks to connect the game to events that happen or might happen to the player in real life. This element was therefore quite straightforward to embed into the game as it was linked to its core concept. We decided to make the player virtually experience scenes that could occur on a normal day to let them make choices that could influence SARS-CoV-2 transmission. We decided to split the game into 4 levels, each related to a specific part of the day (Figures 1 and 2): at home, on the way to work, in communal areas, and in the ward. Including the reflection element has been shown to enhance mental well-being [17] and could thus increase the player’s willingness to adopt desirable behaviors.
- There are two components to the “engagement” element. Social engagement such as that found in multiplayer games is the component that has gained the most popularity in recent history. While such games have shown many positive features, they are also controversial and have been accused of drawing some players away from reality [38]. Given the limited time frame and the goal of our serious game, the inclusion of multiplayer capabilities was not considered relevant. We nevertheless decided to include a social engagement component in the game and to confront the player with the impact some of their choices would have on others. This component was also instrumental in strengthening the reflection element and in keeping the player connected to the real world. The second component of the engagement element is related to the creation of an engaging learning experience and to the concept of “flow” [39]. To avoid boredom and to increase engagement, the challenges should become more difficult as the game progresses. Therefore, simple questions (Figures 3-5) were first asked at the initial level (“at home”), while more complex challenges, such as rebuilding a donning sequence (Figure 6) or choosing the kind of face mask to wear (Figure 7) awaited the player in the last level (“ward”). The main drawback of creating more complex challenges is the risk of generating anxiety and frustration. To avoid these negative effects, feedback mechanisms were used extensively [32].
- The “choice” element relates to the autonomy the player has within the game. Giving the player the ability to make meaningful choices reinforces the player’s autonomy and the feeling of being responsible for their actions. We therefore decided to let players make choices that IPC specialists would disapprove, and to experience (at least virtually) the consequences such potentially harmful choices might have.
- “Information” means providing the player with key concepts to help them understand the reasons behind the serious game, rather than just providing them with points or rewards, which could prove ineffective or even harmful in the long run [40]. Giving players accurate and reliable information ultimately leads to the appropriation of key concepts and generates a positive mental image by generating a feeling of “mastery” regarding these concepts [17]. We therefore included the most recent and evidence-based elements regarding SARS-CoV-2 symptoms and transmission within the game [41-46].
- The “play” element is perhaps the hardest to define, and there are many and sometimes conflicting definitions of this element. We have elected to use Nicholson’s approach [30] in which play is defined as “the freedom to explore and fail within boundaries.” We therefore decided to let players have a certain degree of freedom and to make choices, which may ultimately result in a “game over” situation. The player would then be given the opportunity either to restart the level or to trade tokens to resume the game.
- Exposition means creating a meaningful narrative in the serious game. Engaging the player into the serious game requires capturing their interest and curiosity while still permitting them to make their own choices. To create such a narrative, we decided that the player would virtually go through the steps they usually undertake on a daily basis, and to make choices that may lead to the contamination of patients or of fellow HCWs.

Audio elements were not considered necessary as many hospital employees will play the serious game on institutional computers and will not be able to activate the sound. Attractive graphics adapted to the target population were however deemed essential to increase engagement [47,48].

#### Acquisition Objectives

The IPC specialists defined 10 acquisition objectives according to their field observations. Table 1 summarizes these objectives, the RECIPE element to which they are linked, and gives implementation examples.

**Table 1:** Acquisition objectives, RECIPE (R=reflection, EN=engagement, C=choice, I=information, P=play, and EX=exposition) elements, and implementation examples.

Acquisition objective	RECIPE elements	Implementation
Avoid going to work when potentially infectious	R, EN, C, P, EX	Figures 5,8, 9
Recognize COVID-19 symptoms	I	Figure 10
Recognize that HCWs^a^ have a higher probability of being contaminated by a colleague than by a patient	R, EN, I, EX	Figure 11
Recognize that even asymptomatic carriers can be contagious	EN, I, EX	Figure 12
Recall safety measures in the working environment	R, I, EX	Figures 13 and 14
Recall the correct way of using a face mask	R, I, EX	Figure 15
Recall the correct donning sequence	R, EN, I, EX	Figure 6
Identify when an N95 mask should be worn	R, C, I, EX	Figure 7
Identify when eye protection should be worn	R, C, I, EX	Figure 16
Identify when gloves should be worn and changed	R, C, I, EX	Figure 17

^a^HCW: health care worker.

**Figure 8:**
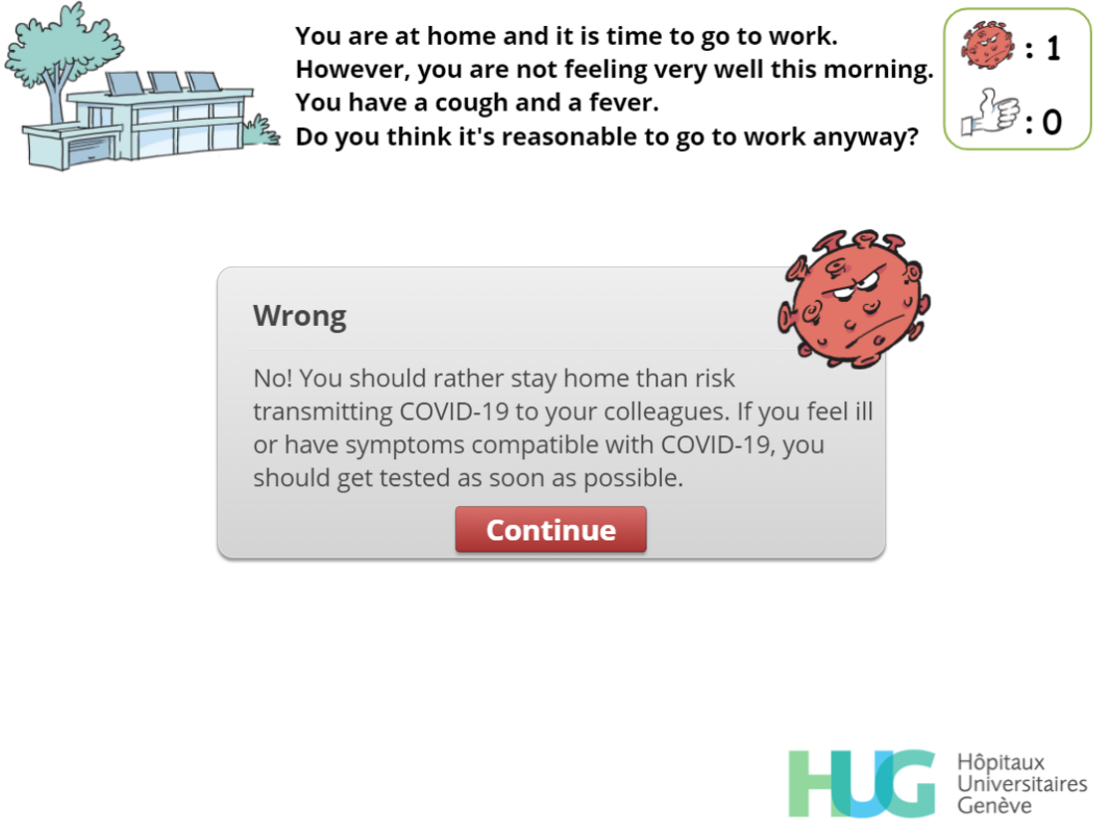
Feedback. The player has chosen to go to work despite the symptoms and is now told they should have undergone a test to screen for SARS-CoV-2 infection. The virus count (top right) has increased accordingly.

**Figure 9:**
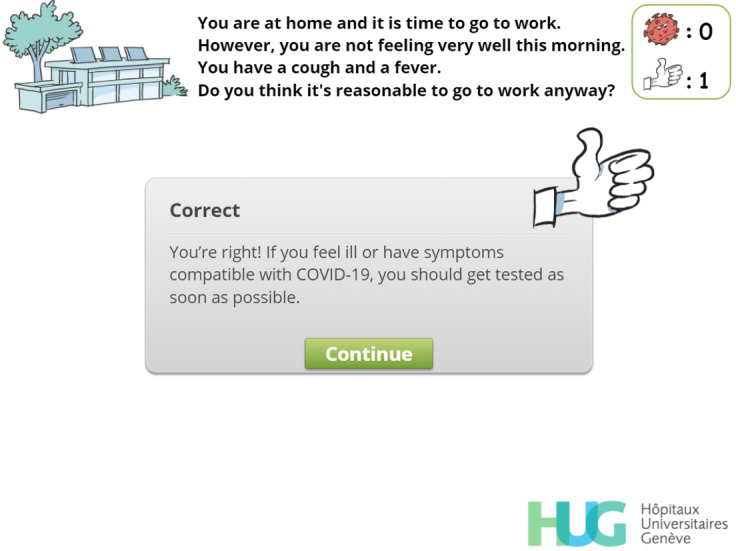
Feedback. The player has chosen not to go to work, and this choice is encouraged by a positive message and by a "thumbs-up" image. The "thumbs-up" count (top right) has increased accordingly.

**Figure 10:**
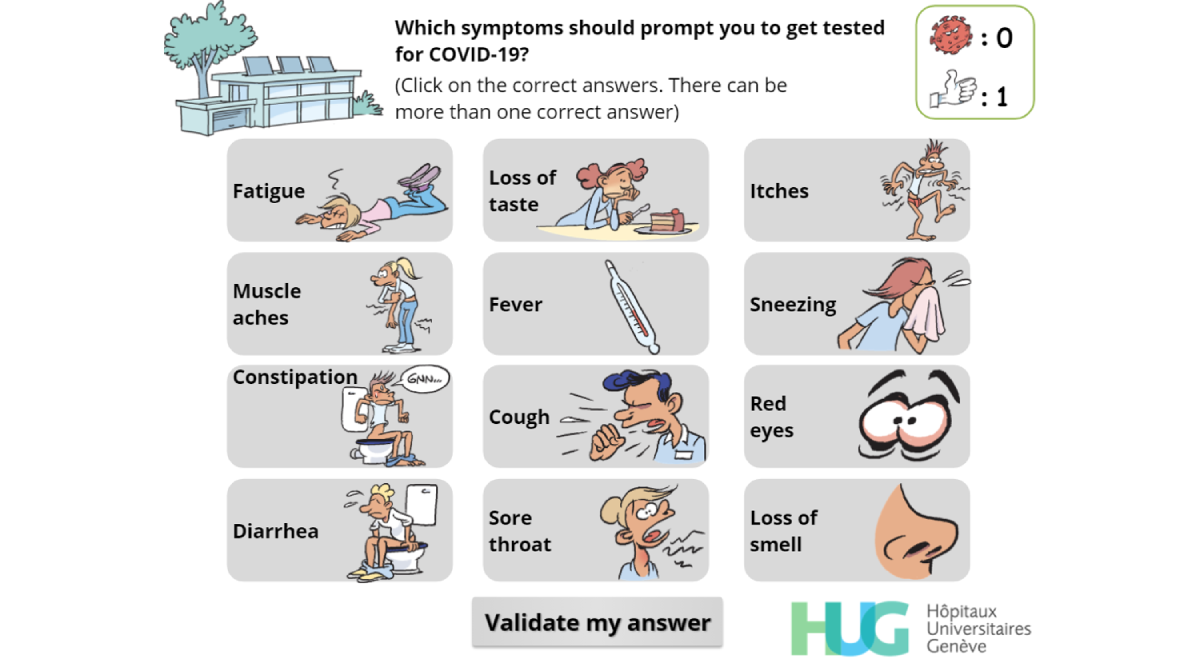
Multiple-choice interaction. The player has to identify the symptoms that should prompt a COVID-19 screening test.

**Figure 11:**
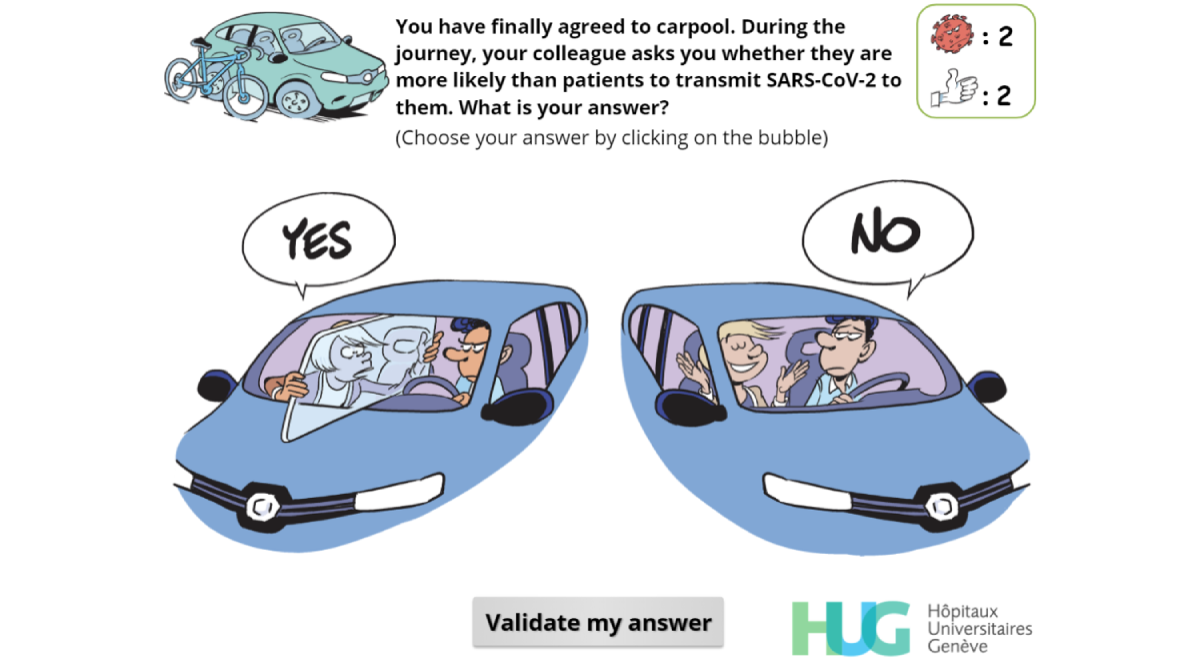
Simple-choice interaction. The player has not selected any option and cannot click on “Validate my answer” for the moment.

**Figure 12:**
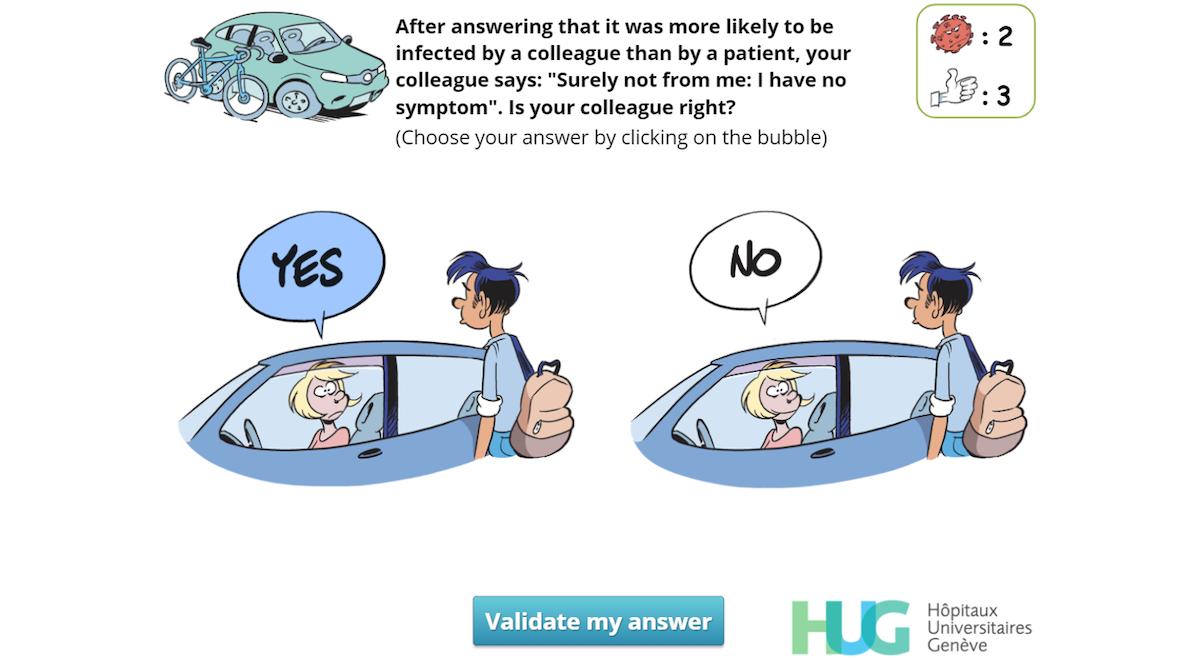
Simple-choice interaction. The player has already clicked on “Yes” and the “Validate my answer” button can now be clicked.

**Figure 13:**
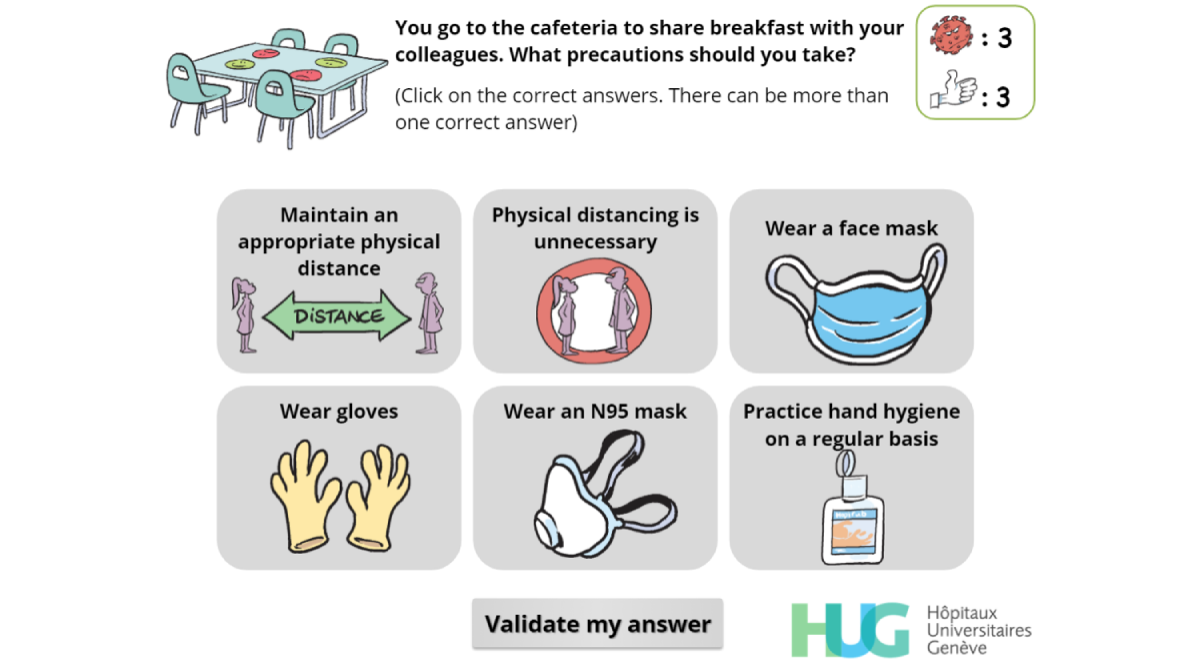
Multiple-choice interaction. The player has to identify the safety precautions that must be taken at work, including at the cafeteria.

**Figure 14:**
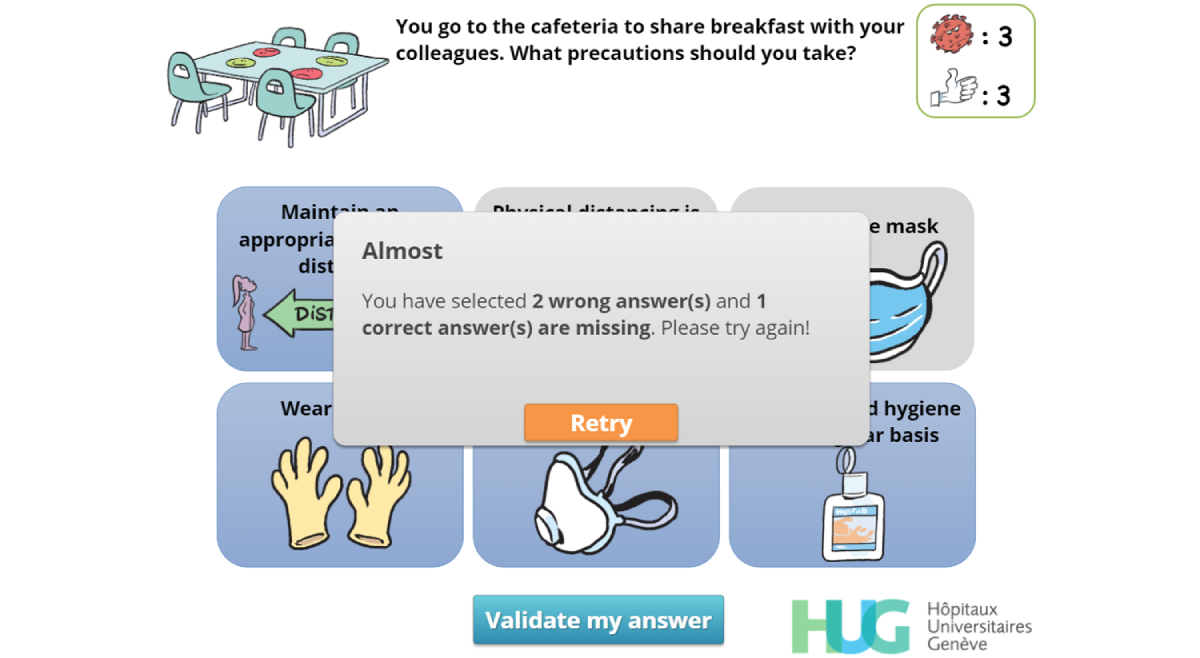
Feedback. The player has failed to choose the expected answers at the first try and can click on “Retry” (retry) to have a second (and last) chance. The number of wrong answers is provided along with the number of correct answers missing from the original attempt.

**Figure 15:**
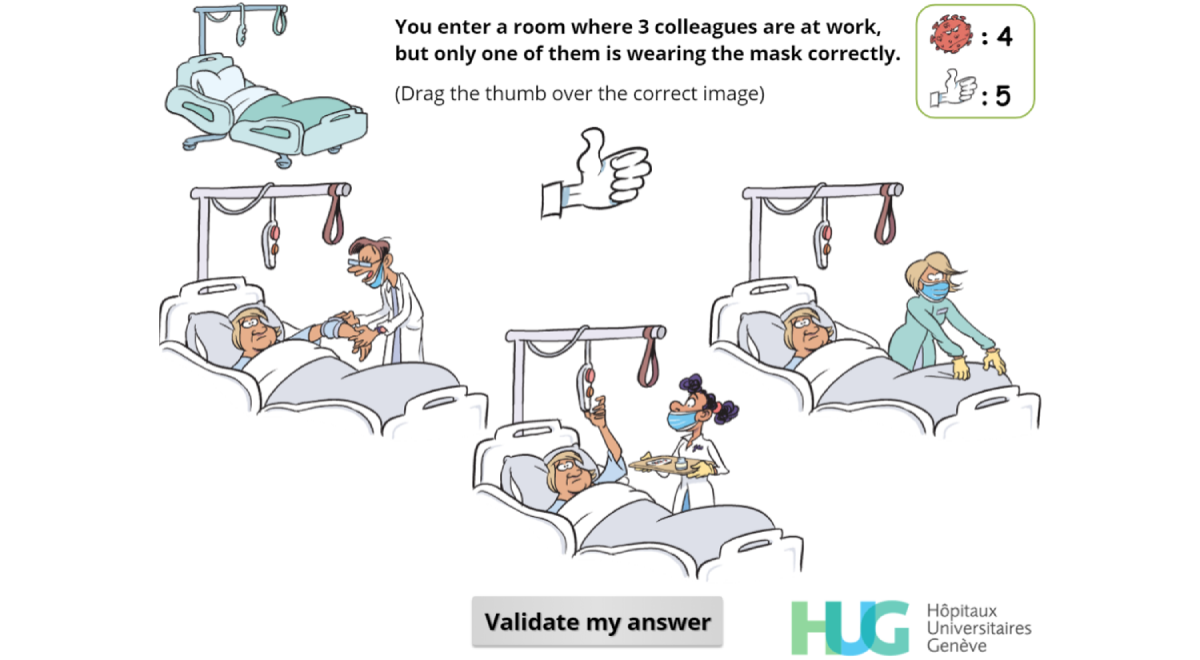
Simple-choice interaction. The player must drag the “thumbs-up” on the image of the only colleague correctly wearing a face mask.

**Figure 16:**
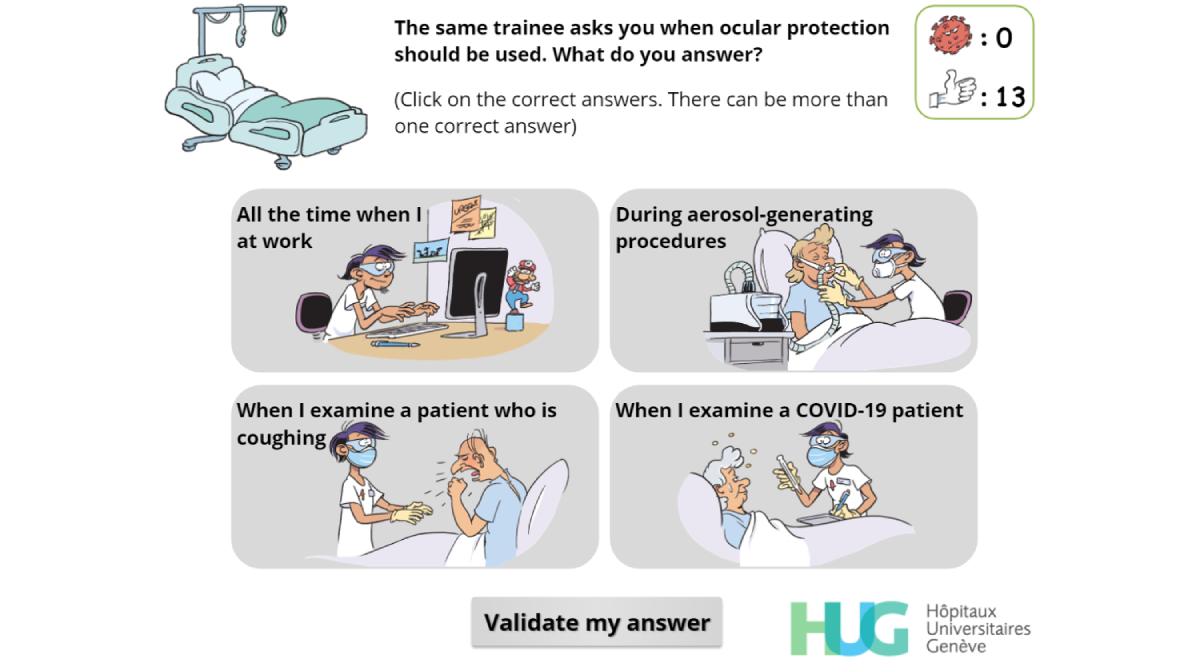
Multiple-choice interaction. The player must choose the situations in which eye protection should be worn.

**Figure 17:**
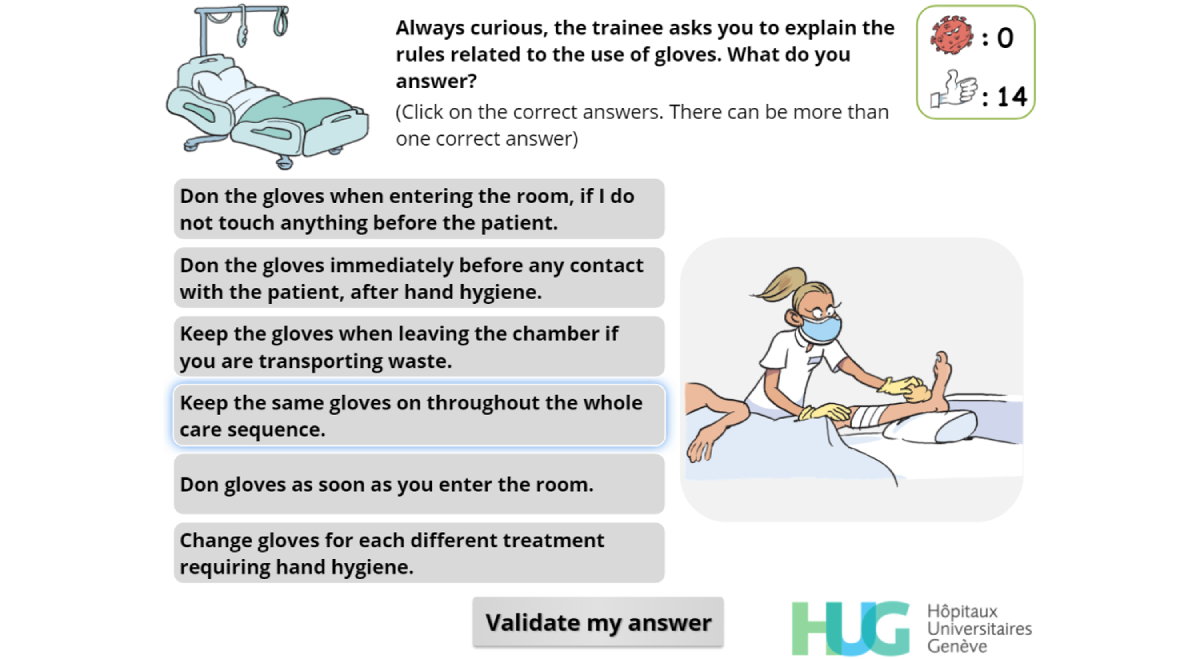
Multiple-choice interaction. The player must choose in which situations they should wear or change gloves. Illustrations change as the cursor hovers over the buttons on the left.

**Figure 18:**
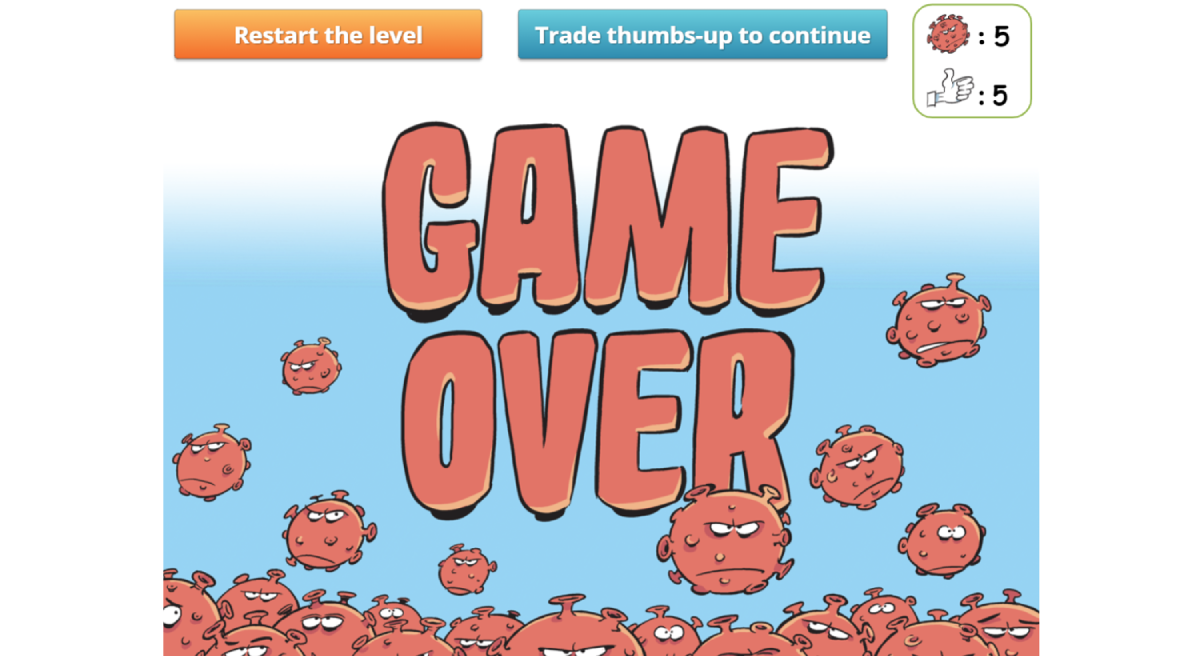
Game over screen. The player can choose to exchange their thumbs-up for viruses (1:1) or to restart the level and decrease the virus count by 2.

To keep track of the player’s choices and to strengthen IPC messages, a virus counter was created. Whenever the player selects an answer that can lead to a possible contamination, a red virus image appears, and the virus count rises incrementally. Conversely, every time the player selects an answer that matches a desirable behavior, a positive token in the form of a “thumbs-up” image is awarded. If the player reaches a total of 5 viruses, a “game over” screen (Figure 18) appears. The user can then choose to decrease the virus count by exchanging “thumbs-up” tokens against viruses or to restart the level. We decided that the players should only restart the level, and not the entire game, in order to provide a more positive feedback and to avoid generating frustration. Moreover, a positive message is displayed at the end of each of the 4 levels. These messages are designed to strengthen the player’s motivation to comply with IPC guidelines and are not influenced by the virus count.

Feedback mechanisms were used extensively and systematically in this serious game [32]. After a simple-choice interaction, the expected answer is immediately provided to the player along with an explanatory message (Figures 8 and 9). When multiple choice interactions are involved, players are allowed to adapt their answer if they were initially wrong. To help them find the correct answers, the number of wrong answers is provided along with the number of correct answers missing from their initial choice (Figure 14).

#### Game Development and Iterations

We first created a storyboard using PowerPoint 16 (Microsoft Corp). This storyboard, split into 4 game levels, was refined through multiple iterations until we deemed it advanced enough to begin the actual game development. A common and simple terminology was used to suit the broad target audience. We chose to develop the actual game under Articulate Storyline 3 (Articulate Global). This software is easy to use, embeds many elements that are essential for creating interactive content and serious games, and allows developers to export their creations to many different platforms using HTML5.

The first version of the game was developed using stick figures created by PresenterMedia (Eclipse Digital Imaging Inc). Figure 1 shows the welcome screen of this first version, and Figure 3 gives an example of a simple-choice interaction.

Although developers and IPC specialists found this first version to be both adequate and usable after having been refined through multiple iterations, they felt that it resembled a gamified e-learning module rather than a serious game. We therefore hired a well-known Swiss graphic designer to help us enhance the graphical aspects of the game and create a unique atmosphere. First, sketches in line with the multiple iterations concept of the SERES framework were produced by the graphic designer. These sketches were revised, adapted, and finally validated. They were then transformed into actual images that were embedded in the serious game. Figure 2 shows the updated (and final) version of the welcome screen, and Figure 4 displays the updated version a simple-choice interaction.

#### Game Validation

Each iteration of the game was tested by at least 3 authors whose feedback was systematically assessed and, when relevant and feasible, integrated into the next iteration of the game. The final version was validated by all the authors and by IPC specialists, some of whom act as consultants for the World Health Organization. Apart from the authors, all of whom are part of the medical or nursing staff, the game was tested by nonclinical staff from the Geneva University Hospitals training center before its release. These testing sessions showed that the game takes 15 minutes to complete on average.

The heuristic evaluation procedure described by Davids et al [49] was used to screen for usability issues. This led to the addition of blinking images in the welcome screen to facilitate identification of interactive elements.

#### Game Availability

This serious game can be freely accessed on the internet [50]. The corresponding author can be contacted to obtain a SCORM (shareable content object reference model) package of the game. We decided to make this package available to facilitate results and completion tracking on most current learning management system. This will allow for the tracking of completion and, on recent platforms, to gather detailed results down to the level of individual questions.

## Discussion

### Principal Findings

The first 3 stages of the SERES framework were successfully used to create the “Escape COVID-19” serious game. The content created meets the definition given by Gentry et al [20] in a recent systematic review (ie, a game created “for the serious purpose of providing health professions education via a digital device”). The boundaries separating serious games from other gamified educational tools are not easy to define [51]. Using the definition Payne et al [52] published in 2015, “Escape COVID-19” qualifies as a serious game as it primarily intends to educate rather than entertain, contains a specific narrative, and includes the possibility of failure. In the context of the COVID-19 pandemic, new HCWs are being quickly recruited to face increasing health care demand [53]. Providing them regular face to face training in IPC is particularly challenging in terms of resources. Distance learning through serious games and other e-learning modalities offers an innovative and cost-effective alternative that can reach a large target population while avoiding the logistical challenges associated with face to face training.

Since HCWs perceive a higher likelihood of contracting COVID-19 than the general population [54] and as fatality rates have been shown to be substantial in this population [55,56], the impact of this serious game could be significant. Distance learning mechanisms have indeed been shown to be of particular value in the context of the current pandemic [57,58]. Still, there are many different e-learning modalities [59,60], and selecting the adequate modality to achieve the intended goal can be challenging. Our gamified e-learning module, which was intended to teach the adequate use of PPE to prehospital personnel, was found to be both useful and satisfactory by paramedics. It however failed to yield significant improvements regarding knowledge acquisition in this population [61]. Conversely, this e-learning modality significantly improved knowledge acquisition in student paramedics who were actively working in an ambulance service at the time of the study [62]. In both populations, however, this gamified module failed to enhance the acquisition of complicated procedures such as PPE donning or doffing [61,62]. Nevertheless, as the goal of this serious game is to promote and strengthen safe behaviors rather than to teach new content to HCWs, it might still prove effective in improving the application of IPC guidelines.

Though we chose to use Nicholson’s RECIPE mnemonic for meaningful gamification [30], we were unable to apply it to its fullest extent. Indeed, the play element was only used in connection with one out of 10 acquisition objectives. Given the goal and design of this serious game, letting the player freely roam in a virtual environment was not an option. Moreover, we elected to force the player to follow a predetermined path, starting at home and ending in the ward. We made this choice to comply with the “flow” component of the engagement element [39]. When designing a serious game, using this component means that challenges should get harder as the player progresses. In the first level (at home), the challenges were relatively easy and the choice element was central. Conversely, in the last level, the player was asked to rebuild a complicated donning sequence and to make difficult choices regarding the kind of PPE that should be worn in specific situations. Therefore, forcing the player to follow a chronological order was relevant in this regard.

### Limitations

The main limitation of this study is the current lack of evidence to support the use of this serious game. A longitudinal trial assessing the evolution of the rate of SARS-CoV-2 contamination in HCWs and in other hospital employees should be carried out to help assess the impact of this serious game. Assessing the added value of specific elements might however prove difficult, and multiple confounding factors (progressive dissemination of new guidelines, natural and unforeseeable evolution of the pandemic, etc) will make this more difficult.

Since knowledge regarding SARS-CoV-2 transmission mechanisms is evolving quickly, this serious game could be obsolete in a matter of months or less. This limitation is however relative as the platform used to develop this serious game is quite flexible and should allow us to rapidly update the game.

Finally, even though most screenshots are displayed in English, the original game was developed in French and the English translation has not been completed yet. It should however be completed by the end of 2020.

### Future Work

While the creation of this serious game was considered successful, we cannot be certain of its actual impact on IPC practices and behavior. The SCORM package is available to any researcher or institution willing to deploy it on a learning management system, whether for research purposes or simply to make it available as an additional educational tool.

Following the release of version 2.08, the Geneva University Hospitals have deployed Escape COVID-19 and made it available to their 13,000 employees, including medical, nursing, and administrative and support staff. In the wake of this deployment, the Geneva Directorate of Health requested us to make this serious game available to nursing home employees. Indeed, long-term care facilities have been severely hit by the COVID-19 pandemic [46,63-65] and motivating their employees to enhance their IPC practices could be of significant benefit [66]. We naturally agreed and took this opportunity to initiate a web-based, triple-blinded randomized controlled trial [67]. The aim of this study is to determine whether Escape COVID-19 can enhance the intention to change IPC practices in nursing home employees.

### Conclusion

The SERES framework was successfully used to create “Escape COVID-19,” a serious game designed to promote safe behaviors among HCWs and other hospital employees during the COVID-19 pandemic. The impact of this game should now be assessed through a longitudinal trial.

## Abbreviations

e-learning: electronic learning
HCW: health care worker
IPC: infection prevention and control
MeSH: Medical Subject Headings
PPE: personal protective equipment
RECIPE: reflection, engagement, choice, information, play, exposition
SCORM: shareable content object reference model
